# 3-(Adamantan-1-yl)-4-ethyl-1*H*-1,2,4-triazole-5(4*H*)-thione

**DOI:** 10.1107/S1600536812014407

**Published:** 2012-04-13

**Authors:** Ali A. El-Emam, Nasser R. El-Brollosy, Hazem A. Ghabbour, Ching Kheng Quah, Hoong-Kun Fun

**Affiliations:** a Department of Pharmaceutical Chemistry, College of Pharmacy, King Saud University, Riyadh 11451, Saudi Arabia; b X-ray Crystallography Unit, School of Physics, Universiti Sains Malaysia, 11800 USM, Penang, Malaysia

## Abstract

In the title compound, C_14_H_21_N_3_S, the 1,2,4-triazole ring is nearly planar, with a maximum deviation of 0.003 (4) Å. In the crystal, mol­ecules are linked into inversion dimers by pairs of N—H⋯S hydrogen bonds.

## Related literature

For the biological activity of adamantane derivatives, see: Al-Omar *et al.* (2010 ▶); Al-Deeb *et al.* (2006 ▶); El-Emam *et al.* (2004 ▶); Kadi *et al.* (2007 ▶, 2010 ▶); Vernier *et al.* (1969 ▶). For the synthesis of the title compound, see: El-Emam & Ibrahim (1991 ▶). For related structures of adamantane derivatives, see: Almutairi *et al.* (2012 ▶); Al-Tamimi *et al.* (2010 ▶); Rouchal *et al.* (2010 ▶); Wang *et al.* (2011 ▶); Al-Abdullah *et al.* (2012 ▶). For standard bond-length data, see: Allen *et al.* (1987 ▶).
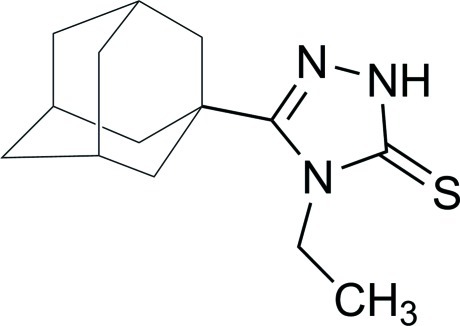


## Experimental

### 

#### Crystal data

C_14_H_21_N_3_S
*M* *_r_* = 263.40
Monoclinic, 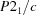

*a* = 13.8329 (7) Å
*b* = 7.3107 (4) Å
*c* = 17.5302 (12) Å
β = 128.157 (4)°
*V* = 1393.99 (16) Å^3^
*Z* = 4
Cu *K*α radiation
μ = 1.94 mm^−1^
*T* = 296 K
0.58 × 0.12 × 0.05 mm

#### Data collection

Bruker SMART APEXII CCD area-detector diffractometer
Absorption correction: multi-scan (*SADABS*; Bruker, 2009 ▶) *T*_min_ = 0.228, *T*_max_ = 0.906
8009 measured reflections
2448 independent reflections
1632 reflections with *I* > 2σ(*I*)
*R*_int_ = 0.093

#### Refinement

*R*[*F* ^2^ > 2σ(*F* ^2^)] = 0.062
*wR*(*F* ^2^) = 0.167
*S* = 1.13
2448 reflections
168 parameters
H atoms treated by a mixture of independent and constrained refinement
Δρ_max_ = 0.19 e Å^−3^
Δρ_min_ = −0.26 e Å^−3^

### 

Data collection: *APEX2* (Bruker, 2009 ▶); cell refinement: *SAINT* (Bruker, 2009 ▶); data reduction: *SAINT*; program(s) used to solve structure: *SHELXTL* (Sheldrick, 2008 ▶); program(s) used to refine structure: *SHELXTL*; molecular graphics: *SHELXTL*; software used to prepare material for publication: *SHELXTL* and *PLATON* (Spek, 2009 ▶).

## Supplementary Material

Crystal structure: contains datablock(s) global, I. DOI: 10.1107/S1600536812014407/is5108sup1.cif

Structure factors: contains datablock(s) I. DOI: 10.1107/S1600536812014407/is5108Isup2.hkl

Supplementary material file. DOI: 10.1107/S1600536812014407/is5108Isup3.cml

Additional supplementary materials:  crystallographic information; 3D view; checkCIF report

## Figures and Tables

**Table 1:** Hydrogen-bond geometry (Å, °)

*D*—H⋯*A*	*D*—H	H⋯*A*	*D*⋯*A*	*D*—H⋯*A*
N2—H1*N*2⋯S1^i^	0.88 (4)	2.47 (4)	3.338 (4)	170 (4)

Symmetry code: (i) 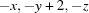.

**Fig. 1.**
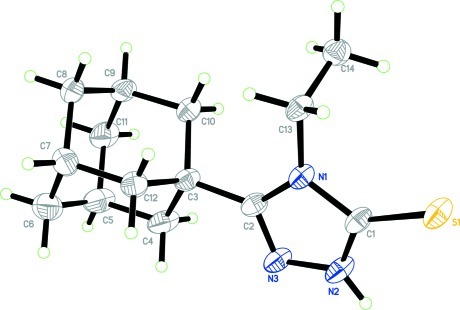
The molecular structure of the title compound, showing 30% probability displacement ellipsoids for non-H atoms.

**Fig. 2.**
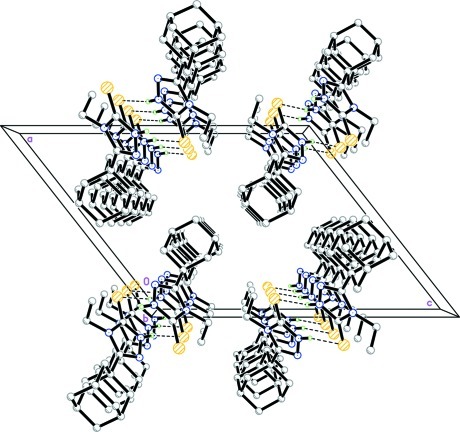
A packing diagram of the title compound, viewed along the b axis. H atoms not involved in hydrogen bonds (dashed lines) have been omitted for clarity.

## supplementary crystallographic information

### Comment

Derivatives of adamantane have long been known for their diverse biological activities including antiviral activity against the influenza (Vernier *et al.*, 1969) and HIV viruses (El-Emam *et al.* 2004). Moreover, adamantane derivatives were recently reported to exhibit marked antibacterial activity (Kadi *et al.*, 2007, 2010). In an earlier publication, we reported the synthesis and potent anti-inflammatory and analgesic activities of a series of 5-(1-adamantyl)-4-substituted-4*H*-1,2,4-triazole-3-thiols and related derivatives including the title compound (El-Emam & Ibrahim, 1991).

In the title molecule (Fig. 1), the 1,2,4-triazole ring (N1–N3/C1/C2) is nearly planar with a maximum deviation of 0.003 (4)) Å at atom C1. Bond lengths (Allen *et al.*, 1987) and angles are within normal ranges and are comparable to related structures (Almutairi *et al.*, 2012; Al-Tamimi *et al.*, 2010; Rouchal *et al.*, 2010; Wang *et al.*, 2011; Al-Abdullah *et al.*, 2012).

In the crystal (Fig. 2), molecules are linked into inversion dimers by pairs of intermolecular N2—H2A···S1 hydrogen bonds (Table 1).

### Experimental

A mixture of adamantane-1-carbohydrazide (1.94 g, 0.01 mol), ethyl isothiocyanate (0.87 g, 0.01 mol), in ethanol (10 ml) was heated under reflux with stirring for one hour and the solvent was distilled off *in vacuo*. Aqueous sodium hydroxide solution (10%, 15 ml) was added to the residue and the mixture was heated under reflux for 2 h, then filtered hot. On cooling, the mixture was acidified with hydrochloric acid and the precipitated crude product was filtered, washed with water, dried and crystallized from aqueous ethanol to yield 2.24 g (85%) of the title compound (C_14_H_21_N_3_S) as colorless crystals. *M*.*p*.: 210-212 °C. ^1^H NMR (CDCl_3_, 500.13 MHz): δ 1.36 (t, 3H, C*H*_3_CH_2_, *J* = 7.0 Hz), 1.73 (s, 6H, Adamantane-H), 1.99 (m, 6H, Adamantane-H), 2.06 (s, 3H, Adamantane-H), 4.19 (q, 2H, CH_3_C*H*_2_), 11.60 (br. s, 1H, NH). ^13^C NMR (CDCl_3_, 125.76 MHz): δ 13.99 (CH_3_), 27.91, 35.48, 36.27, 39.75 (Adamantane-C), 41.17 (CH_2_), 158.04 (C=N), 167.25 (C=S).

### Refinement

Atom H1N2 was located in a difference Fourier map and refined freely [N—H = 0.87 (4) Å]. The remaining hydrogen atoms were positioned geometrically (C—H = 0.96–0.98 Å) and were refined using a riding model, with *U*_iso_(H) = 1.2 or 1.5*U*_eq_(C). A rotating group model was applied to the methyl group.

### Crystal data

**Table:**

C_14_H_21_N_3_S	*F*(000) = 568
*M**_r_* = 263.40	*D*_x_ = 1.255 Mg m^−^^3^
Monoclinic, *P*2_1_/*c*	Cu *K*α radiation, λ = 1.54178 Å
Hall symbol: -P 2ybc	Cell parameters from 592 reflections
*a* = 13.8329 (7) Å	θ = 4.1–66.5°
*b* = 7.3107 (4) Å	µ = 1.94 mm^−^^1^
*c* = 17.5302 (12) Å	*T* = 296 K
β = 128.157 (4)°	Needle, colourless
*V* = 1393.99 (16) Å^3^	0.58 × 0.12 × 0.05 mm
*Z* = 4	

### Data collection

**Table:**

Bruker SMART APEXII CCD area-detector diffractometer	2448 independent reflections
Radiation source: fine-focus sealed tube	1632 reflections with *I* > 2σ(*I*)
Graphite monochromator	*R*_int_ = 0.093
φ and ω scans	θ_max_ = 67.5°, θ_min_ = 4.1°
Absorption correction: multi-scan (*SADABS*; Bruker, 2009)	*h* = −16→16
*T*_min_ = 0.228, *T*_max_ = 0.906	*k* = −8→6
8009 measured reflections	*l* = −20→19

### Refinement

**Table:**

Refinement on *F*^2^	Primary atom site location: structure-invariant direct methods
Least-squares matrix: full	Secondary atom site location: difference Fourier map
*R*[*F*^2^ > 2σ(*F*^2^)] = 0.062	Hydrogen site location: inferred from neighbouring sites
*wR*(*F*^2^) = 0.167	H atoms treated by a mixture of independent and constrained refinement
*S* = 1.13	*w* = 1/[σ^2^(*F*_o_^2^) + (0.0553*P*)^2^ + 1.0409*P*] where *P* = (*F*_o_^2^ + 2*F*_c_^2^)/3
2448 reflections	(Δ/σ)_max_ = 0.001
168 parameters	Δρ_max_ = 0.19 e Å^−^^3^
0 restraints	Δρ_min_ = −0.26 e Å^−^^3^

### Special details

**Table:**

Geometry. All esds (except the esd in the dihedral angle between two l.s. planes) are estimated using the full covariance matrix. The cell esds are taken into account individually in the estimation of esds in distances, angles and torsion angles; correlations between esds in cell parameters are only used when they are defined by crystal symmetry. An approximate (isotropic) treatment of cell esds is used for estimating esds involving l.s. planes.
Refinement. Refinement of F^2^ against ALL reflections. The weighted R-factor wR and goodness of fit S are based on F^2^, conventional R-factors R are based on F, with F set to zero for negative F^2^. The threshold expression of F^2^ > 2sigma(F^2^) is used only for calculating R-factors(gt) etc. and is not relevant to the choice of reflections for refinement. R-factors based on F^2^ are statistically about twice as large as those based on F, and R- factors based on ALL data will be even larger.

### Fractional atomic coordinates and isotropic or equivalent isotropic displacement parameters (Å^2^)

**Table:**

	*x*	*y*	*z*	*U*_iso_*/*U*_eq_	
S1	−0.10923 (11)	0.88672 (13)	0.05119 (8)	0.0602 (4)	
N1	0.0606 (3)	0.6128 (4)	0.1520 (2)	0.0452 (7)	
N2	0.0957 (4)	0.7839 (4)	0.0738 (3)	0.0591 (10)	
N3	0.1903 (3)	0.6609 (4)	0.1203 (2)	0.0571 (9)	
C1	0.0152 (4)	0.7606 (4)	0.0913 (3)	0.0490 (10)	
C2	0.1677 (4)	0.5561 (5)	0.1684 (3)	0.0479 (9)	
C3	0.2544 (4)	0.4078 (5)	0.2347 (3)	0.0505 (9)	
C4	0.3537 (4)	0.3859 (5)	0.2218 (4)	0.0733 (13)	
H4A	0.3954	0.5018	0.2345	0.088*	
H4B	0.3158	0.3509	0.1554	0.088*	
C5	0.4475 (5)	0.2398 (6)	0.2914 (5)	0.0821 (15)	
H5A	0.5104	0.2284	0.2822	0.099*	
C6	0.5089 (5)	0.2972 (7)	0.3961 (5)	0.0953 (18)	
H6A	0.5695	0.2068	0.4406	0.114*	
H6B	0.5504	0.4136	0.4096	0.114*	
C7	0.4113 (4)	0.3141 (6)	0.4098 (3)	0.0710 (12)	
H7A	0.4502	0.3481	0.4771	0.085*	
C8	0.3435 (4)	0.1329 (5)	0.3865 (3)	0.0660 (12)	
H8A	0.2810	0.1455	0.3951	0.079*	
H8B	0.4009	0.0391	0.4307	0.079*	
C9	0.2836 (4)	0.0760 (5)	0.2820 (3)	0.0587 (11)	
H9A	0.2415	−0.0414	0.2682	0.070*	
C10	0.1908 (4)	0.2214 (5)	0.2139 (3)	0.0524 (10)	
H10A	0.1279	0.2312	0.2223	0.063*	
H10B	0.1512	0.1862	0.1473	0.063*	
C11	0.3813 (5)	0.0576 (6)	0.2681 (4)	0.0781 (14)	
H11A	0.3433	0.0231	0.2016	0.094*	
H11B	0.4397	−0.0369	0.3106	0.094*	
C12	0.3189 (4)	0.4605 (5)	0.3421 (3)	0.0595 (11)	
H12A	0.3607	0.5768	0.3564	0.071*	
H12B	0.2579	0.4739	0.3523	0.071*	
C13	−0.0034 (4)	0.5359 (5)	0.1871 (3)	0.0486 (9)	
H13A	−0.0375	0.6350	0.2006	0.058*	
H13B	0.0553	0.4710	0.2474	0.058*	
C14	−0.1058 (4)	0.4064 (5)	0.1148 (3)	0.0577 (10)	
H14A	−0.1485	0.3664	0.1389	0.087*	
H14B	−0.0717	0.3024	0.1056	0.087*	
H14C	−0.1622	0.4682	0.0540	0.087*	
H1N2	0.089 (3)	0.868 (5)	0.035 (3)	0.063 (12)*	

### Atomic displacement parameters (Å^2^)

**Table:**

	*U*^11^	*U*^22^	*U*^33^	*U*^12^	*U*^13^	*U*^23^
S1	0.0812 (8)	0.0444 (6)	0.0664 (7)	0.0174 (5)	0.0513 (6)	0.0157 (4)
N1	0.060 (2)	0.0367 (15)	0.0490 (17)	0.0075 (14)	0.0389 (16)	0.0066 (12)
N2	0.088 (3)	0.0436 (19)	0.069 (2)	0.0136 (17)	0.060 (2)	0.0182 (16)
N3	0.076 (2)	0.0419 (17)	0.074 (2)	0.0115 (16)	0.057 (2)	0.0146 (15)
C1	0.071 (3)	0.0329 (18)	0.048 (2)	0.0057 (17)	0.039 (2)	0.0031 (15)
C2	0.064 (3)	0.0383 (19)	0.053 (2)	0.0010 (17)	0.042 (2)	0.0002 (15)
C3	0.059 (3)	0.040 (2)	0.064 (2)	0.0053 (17)	0.043 (2)	0.0074 (16)
C4	0.086 (3)	0.051 (2)	0.120 (4)	0.012 (2)	0.082 (3)	0.020 (2)
C5	0.074 (3)	0.062 (3)	0.140 (5)	0.022 (2)	0.080 (4)	0.029 (3)
C6	0.061 (3)	0.071 (3)	0.127 (5)	0.005 (3)	0.045 (3)	0.025 (3)
C7	0.055 (3)	0.064 (3)	0.068 (3)	−0.004 (2)	0.025 (2)	0.005 (2)
C8	0.053 (3)	0.057 (3)	0.070 (3)	0.007 (2)	0.029 (2)	0.023 (2)
C9	0.061 (3)	0.037 (2)	0.078 (3)	0.0047 (18)	0.043 (2)	0.0093 (18)
C10	0.062 (3)	0.044 (2)	0.055 (2)	0.0045 (18)	0.038 (2)	0.0048 (16)
C11	0.081 (3)	0.052 (3)	0.118 (4)	0.022 (2)	0.070 (3)	0.020 (3)
C12	0.060 (3)	0.045 (2)	0.063 (3)	−0.0037 (18)	0.032 (2)	0.0001 (18)
C13	0.065 (3)	0.043 (2)	0.052 (2)	0.0083 (18)	0.043 (2)	0.0071 (16)
C14	0.060 (3)	0.049 (2)	0.067 (3)	−0.0003 (19)	0.041 (2)	−0.0006 (18)

### Geometric parameters (Å, º)

**Table:**

S1—C1	1.677 (4)	C7—C12	1.521 (5)
N1—C1	1.367 (4)	C7—C8	1.526 (5)
N1—C2	1.386 (5)	C7—H7A	0.9800
N1—C13	1.468 (4)	C8—C9	1.530 (6)
N2—C1	1.337 (5)	C8—H8A	0.9700
N2—N3	1.366 (4)	C8—H8B	0.9700
N2—H1N2	0.87 (4)	C9—C10	1.520 (5)
N3—C2	1.312 (4)	C9—C11	1.522 (6)
C2—C3	1.497 (5)	C9—H9A	0.9800
C3—C4	1.533 (5)	C10—H10A	0.9700
C3—C10	1.539 (5)	C10—H10B	0.9700
C3—C12	1.553 (5)	C11—H11A	0.9700
C4—C5	1.535 (6)	C11—H11B	0.9700
C4—H4A	0.9700	C12—H12A	0.9700
C4—H4B	0.9700	C12—H12B	0.9700
C5—C11	1.522 (6)	C13—C14	1.514 (5)
C5—C6	1.530 (7)	C13—H13A	0.9700
C5—H5A	0.9800	C13—H13B	0.9700
C6—C7	1.515 (7)	C14—H14A	0.9600
C6—H6A	0.9700	C14—H14B	0.9600
C6—H6B	0.9700	C14—H14C	0.9600
			
C1—N1—C2	108.5 (3)	C7—C8—C9	110.2 (3)
C1—N1—C13	121.5 (3)	C7—C8—H8A	109.6
C2—N1—C13	130.0 (3)	C9—C8—H8A	109.6
C1—N2—N3	113.9 (3)	C7—C8—H8B	109.6
C1—N2—H1N2	124 (3)	C9—C8—H8B	109.6
N3—N2—H1N2	122 (3)	H8A—C8—H8B	108.1
C2—N3—N2	104.3 (3)	C10—C9—C11	109.8 (3)
N2—C1—N1	103.4 (3)	C10—C9—C8	108.7 (3)
N2—C1—S1	128.2 (3)	C11—C9—C8	109.8 (4)
N1—C1—S1	128.3 (3)	C10—C9—H9A	109.5
N3—C2—N1	109.9 (3)	C11—C9—H9A	109.5
N3—C2—C3	121.8 (3)	C8—C9—H9A	109.5
N1—C2—C3	128.2 (3)	C9—C10—C3	110.6 (3)
C2—C3—C4	108.8 (3)	C9—C10—H10A	109.5
C2—C3—C10	113.0 (3)	C3—C10—H10A	109.5
C4—C3—C10	107.9 (3)	C9—C10—H10B	109.5
C2—C3—C12	110.2 (3)	C3—C10—H10B	109.5
C4—C3—C12	108.0 (3)	H10A—C10—H10B	108.1
C10—C3—C12	108.8 (3)	C9—C11—C5	109.0 (3)
C3—C4—C5	110.5 (3)	C9—C11—H11A	109.9
C3—C4—H4A	109.6	C5—C11—H11A	109.9
C5—C4—H4A	109.6	C9—C11—H11B	109.9
C3—C4—H4B	109.6	C5—C11—H11B	109.9
C5—C4—H4B	109.6	H11A—C11—H11B	108.3
H4A—C4—H4B	108.1	C7—C12—C3	110.3 (3)
C11—C5—C6	111.0 (4)	C7—C12—H12A	109.6
C11—C5—C4	108.8 (4)	C3—C12—H12A	109.6
C6—C5—C4	109.4 (4)	C7—C12—H12B	109.6
C11—C5—H5A	109.2	C3—C12—H12B	109.6
C6—C5—H5A	109.2	H12A—C12—H12B	108.1
C4—C5—H5A	109.2	N1—C13—C14	112.5 (3)
C7—C6—C5	108.9 (4)	N1—C13—H13A	109.1
C7—C6—H6A	109.9	C14—C13—H13A	109.1
C5—C6—H6A	109.9	N1—C13—H13B	109.1
C7—C6—H6B	109.9	C14—C13—H13B	109.1
C5—C6—H6B	109.9	H13A—C13—H13B	107.8
H6A—C6—H6B	108.3	C13—C14—H14A	109.5
C6—C7—C12	109.6 (4)	C13—C14—H14B	109.5
C6—C7—C8	110.4 (4)	H14A—C14—H14B	109.5
C12—C7—C8	108.7 (3)	C13—C14—H14C	109.5
C6—C7—H7A	109.4	H14A—C14—H14C	109.5
C12—C7—H7A	109.4	H14B—C14—H14C	109.5
C8—C7—H7A	109.4		
			
C1—N2—N3—C2	0.3 (4)	C11—C5—C6—C7	−59.4 (5)
N3—N2—C1—N1	−0.6 (4)	C4—C5—C6—C7	60.7 (5)
N3—N2—C1—S1	177.7 (3)	C5—C6—C7—C12	−61.3 (5)
C2—N1—C1—N2	0.6 (4)	C5—C6—C7—C8	58.3 (5)
C13—N1—C1—N2	−178.0 (3)	C6—C7—C8—C9	−58.9 (5)
C2—N1—C1—S1	−177.7 (3)	C12—C7—C8—C9	61.4 (5)
C13—N1—C1—S1	3.8 (5)	C7—C8—C9—C10	−61.4 (4)
N2—N3—C2—N1	0.1 (4)	C7—C8—C9—C11	58.8 (4)
N2—N3—C2—C3	−176.4 (3)	C11—C9—C10—C3	−60.1 (4)
C1—N1—C2—N3	−0.4 (4)	C8—C9—C10—C3	60.0 (4)
C13—N1—C2—N3	177.9 (3)	C2—C3—C10—C9	178.9 (3)
C1—N1—C2—C3	175.8 (3)	C4—C3—C10—C9	58.6 (4)
C13—N1—C2—C3	−5.9 (6)	C12—C3—C10—C9	−58.3 (4)
N3—C2—C3—C4	−8.8 (5)	C10—C9—C11—C5	60.7 (5)
N1—C2—C3—C4	175.4 (4)	C8—C9—C11—C5	−58.8 (5)
N3—C2—C3—C10	−128.6 (4)	C6—C5—C11—C9	59.8 (5)
N1—C2—C3—C10	55.6 (5)	C4—C5—C11—C9	−60.7 (5)
N3—C2—C3—C12	109.5 (4)	C6—C7—C12—C3	61.0 (5)
N1—C2—C3—C12	−66.3 (5)	C8—C7—C12—C3	−59.7 (5)
C2—C3—C4—C5	177.9 (4)	C2—C3—C12—C7	−177.4 (3)
C10—C3—C4—C5	−59.2 (5)	C4—C3—C12—C7	−58.7 (4)
C12—C3—C4—C5	58.2 (5)	C10—C3—C12—C7	58.2 (4)
C3—C4—C5—C11	61.2 (5)	C1—N1—C13—C14	82.7 (4)
C3—C4—C5—C6	−60.3 (5)	C2—N1—C13—C14	−95.5 (4)

### Hydrogen-bond geometry (Å, º)

**Table:**

*D*—H···*A*	*D*—H	H···*A*	*D*···*A*	*D*—H···*A*
N2—H1*N*2···S1^i^	0.88 (4)	2.47 (4)	3.338 (4)	170 (4)

Symmetry code: (i) −*x*, −*y*+2, −*z*.
